# Bioengineering cytotoxic T cells to target opportunistic fungal infection

**DOI:** 10.1186/2051-1426-1-S1-P4

**Published:** 2013-11-07

**Authors:** Pappanaicken Kumaresan, Pallavi R Manuri, Nathaniel Albert, Brian Rabinovich, Simon Olivares, Sourindra N Maiti, Helen Huls, Dean A Lee, Dimitrios Kontoyiannis, Laurence J Cooper

**Affiliations:** 1 Department of Pediatrics, MDACC, Houston, TX, USA; 2 Department of Infectious Disease, MDACC, Houston, TX, USA

## 

Clinical-grade T cells are genetically modified ex vivo to express chimeric antigen receptors (CARs) to redirect their specificity to target tumor-associated antigens in vivo. We have developed gene therapy approach to render T cells specific for invasive fungal infections (IFI) due to Aspergillus. We adapted the pattern-recognition receptor Dectin-1 to activate T cells via chimeric CD28 and CD3-zeta (designated D-CAR) upon binding with carbohydrate cell wall in Aspergillus germlings. T cells genetically modified with Sleeping Beauty system to stably express D-CAR were selectively propagated on artificial antigen presenting cells using an approach that is approved by FDA to develop CAR T cells for clinical trials. The D-CAR+ T cells exhibited specificity for beta-1,3-gucan and damaged and thus inhibited hyphal growth of Aspergillus. Treatment of D-CAR+ T cells with steroids did not compromise anti-fungal activity. Thus, we report a clinically-appealing strategy to transfer innate immunity for mycology to cytotoxic T cells.

